# Synthesis and Characterization of Lignin-Derived Porous Materials from *Phyllostachys edulis* (Bamboo Moso) for the Removal of Aromatic Pollutants

**DOI:** 10.1021/acsomega.5c06041

**Published:** 2025-11-17

**Authors:** Andrea Marangon, Elisa Calà, Alessandro Croce, Geo Paul, Giorgio Gatti

**Affiliations:** † Dipartimento per lo Sviluppo Sostenibile e la Transizione Ecologica, 19050 Università degli Studi del Piemonte Orientale, Piazza Sant’Eusebio 5, 13100 Vercelli, Italy; ‡ GEA G.S. s.r.l.s., Piazza S. Eusebio 5, 13100 Vercelli, Italy; § SSD Research Laboratories, Research and Innovation Department (DAIRI), 9263 Azienda Ospedaliero-Universitaria SS. Antonio e Biagio e Cesare Arrigo, Via Venezia 16, 15121 Alessandria, Italy; ∥ Dipartimento di Scienze e Innovazione Tecnologica, 19050 Università degli Studi del Piemonte Orientale, Viale Teresa Michel 11, 15121 Alessandria, Italia

## Abstract

In recent decades, pollution from dyes has increasingly attracted the attention of researchers. For this reason, the development of new materials capable of sequestering this type of pollutant has been extensively studied, especially as research using plant biomass to produce new materials. Considerable attention has been directed toward bamboo moso, which has been the focus of extensive studies. The chemical composition of bamboo culms indicates that cellulose and hemicellulose comprise 65–70%, whereas lignin accounts for 18–30%. In this study, lignin has been extracted from bamboo culms using the hydrothermal methodology under basic conditions. Water-soluble lignin was dispersed in a porogenic organic solvent and polymerized via the Friedel-Craft alkylation reaction. ^13^C CPMAS NMR spectroscopy was used to study the modification of the water-soluble lignin before and after polymerization; the peaks related to the linker reaction were detected and confirmed the successful cross-linking reaction. The biopolymer-composite was also characterized by FTIR spectroscopy to explore the surface functionality and its interaction with the aromatic molecules, using toluene as a model compound. To gain a deeper understanding of the surface properties, N_2_ physisorption analysis was performed at 77 K. To evaluate the material’s capacity to act as an adsorbent for organic dyes, the adsorption kinetics of organic pollutants were simulated using Crystal Violet as a model molecule, tracking the adsorption trends through UV–vis spectroscopy. In this context, the adsorption capacity of Crystal Violet was determined to exceed 98% within the initial 4 h period.

## Graphical abstract

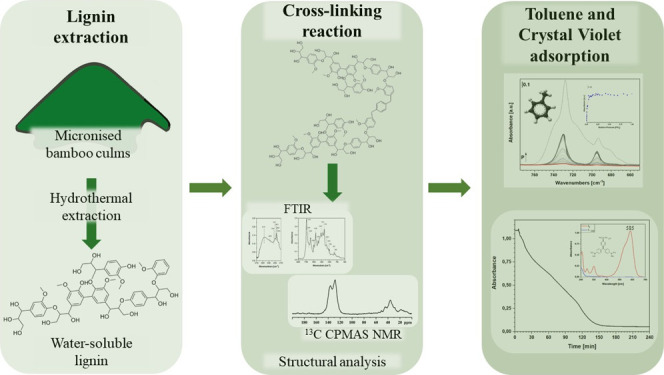

## Introduction

Water pollution has recently emerged as one of the most frequently addressed environmental issues. New pollutants are being continually investigated. Synthetic dyes represent one of the most widespread water pollutants. To maintain their color tones, all synthetic dyes must demonstrate good stability during aging. They possess extensive aromatic structures, high light stability, and moderate solubility in water.^1^

Currently, textile dyeing processes rank among the most impactful industrial activities due to their high water consumption and the substantial volumes of wastewater containing dyes that require disposal. Wastewater from these dyeing processes is not always disposed of properly, resulting in dyes contaminating watercourses, groundwater, and seas. Consequently, various solutions to this issue have been sought over the past few decades. The solutions proposed in the literature primarily emphasize the degradation of dyes using metal catalysts and electrochemical degradation on various adsorbent materials.^1–4^ Among the adsorbent materials proposed in the literature, there are activated carbons with various characteristics (porosity, graphitization, doping with metal cations, conductivity, and quantities of different functional groups), zeolites, silica, metal organic frameworks (MOFs), covalent organic frameworks (COFs), and biomaterials. This latter category encompasses all materials derived from waste biomass, such as cellulose, chitin, chitosan, starch, and lignin.^5–13^

Lignin, a natural polymer identified within the cell walls of plants, plays a critical role in maintaining the structural and physical integrity of these organisms. In addition to its mechanical properties, lignin plays a fundamental role in regulating the continuous flow of water through hydrophobic interactions, which helps prevent damage from fungal and insect infestations.^10,14^ From a chemical standpoint, lignin is composed of a random network of phenylpropionic groups. The monomeric units constituting lignin include coumaryl alcohol, coniferyl alcohol, and sinapyl alcohol. The synthesis of lignin occurs via the radical reaction of these three monomers, resulting in a three-dimensional structure comprising various functional groups, including methoxy, phenolic hydroxyl, aliphatic hydroxyl, and carbonyl groups.^10,14–16^

There has been a notable increase in interest in lignin-based biomaterials in recent years, with research focusing on lignin extraction from plant biomass. The extraction process varies in terms of temperature, pressure, and solvent type. The central aspect of all lignin extraction processes is breaking the β-O-4 ether bonds that link the individual monomers, which is followed by the solubilization of the extracted fragments. Lignin can be extracted from biomass and transformed into biofuels, biocomposites, and bioplastics.^17^ Lignin can also be extracted and converted into various materials that can be used to remove pollutants from water.^18–21^

The most common materials from lignin include biochar, hydrogels, aerogels, nanoparticles, flame-retardant additives, adhesives, and different kinds of coatings. In the case of biochar, hydrogel, and nanoparticles, the high surface area values characterize these materials due to their particle sizes or intrinsic porosity structures.^3,10,22–35^ These materials can undergo functionalization to enhance their affinity for the adsorption of a particular class of compounds. The removal efficiency of synthetic dyes on adsorbent materials is assessed across multiple adsorption cycles, and the amount of dyes removed and subsequently released under appropriate operating conditions is calculated. All the mentioned materials have been extensively investigated in literature, and their adsorbent capacities concerning anionic and cationic dyes are remarkably high (>90%).^10^ Lignin can be extracted from various biomass sources, and the yield varies depending on the initial biomass, the biomass source, and the extraction method used. Numerous lignin extraction methods are documented in the literature, including Kraft lignin, sulfite pulping, soda-based extraction, organosolv extraction, steam explosion, and dilute acid lignin extraction.^36,37^ A less thorough method of preparing materials from lignin involves the repolymerization of lignin extracts. In this method, lignin fragments extracted from biomass can be repolymerized through various routes, such as pH change, solvent extraction, or reaction with molecules that can impart different functionalities to the resulting polymer.^38,39^ Literature proposes different classes of lignin-based materials; in particular, a lot of works are focused on lignin nanoparticles, without any porosity, limiting the specific surface area to the external area of the nanoparticles.^40,41^

Furthermore, lignin and derived materials, mainly nanoparticles and materials derived from the repolymerization of lignin, exhibit not only adsorbent properties but also antibacterial properties on various types of bacteria, such as *Escherichia coli* and *Staphylococcus aureus*.^42^

A lot of different vegetable biomass can be used for lignin extraction. Bamboo is one of the biomass sources that have attracted the most interest in recent years, thanks to its high growth rates, CO_2_ absorption and storage capacity, as well as its ability to absorb heavy metals such as lead.^43,44^

The Friedel–Crafts reaction is one of the most widely used organic reactions. This type involves using Lewis acids as catalysts, such as ZnCl_2_, FeCl_3_, AlCl_3_, and SnCl_4_.^45^ The Friedel–Crafts alkylation reaction is a method for forming carbon–carbon bonds in organic synthesis.^46^ This reaction involves the formation of a carbocationic species following the reaction of a halogenated organic molecule with a Lewis acid. A carbon–carbon bond is then formed when the cationic carbon atom reacts with the carbon of an aromatic ring.^47^ This study utilized lignin extracted from bamboo culms. Bamboo is able to absorb a large amount of CO_2_ from the atmosphere and store it, using photosynthesis, as organic carbon. Water-soluble lignin was dispersed in a porogenic organic solvent and polymerized via the Friedel-Craft alkylation reaction. The resulting biopolymer-composite was characterized using ^13^C CPMAS NMR, FTIR spectroscopy, N_2_ physisorption and thermogravimetric analyses. Finally, the adsorption capacity of biopolymer-composite toward the organic dye, Crystal Violet as a model molecule, from aqueous phase was investigated by means of UV–vis spectroscopy.

## Experimental and Methods

### Lignin Extraction

Lignin was extracted from micronized bamboo (∼100 μm) culms using a 60 mL water solution containing 10 mg of anthraquinone and 4 g of NaOH. The bamboo powder and water solution (in a 1:6 m/m ratio) were placed in an autoclave at 170 °C for 24 h. After this, the solution was cooled to room temperature and then separated from the cellulose solid residue by filtration. The cellulose residue was subsequently washed with deionized water to remove any discolouration.^48^ The lignin solution was subsequently freeze-dried following filtration.

### Synthesis

After extraction, 2 g of extracted lignin was dispersed in 100 mL of 1,2-dichloroethane (Sigma-Aldrich, 107-06-2), maintaining the temperature at 60 °C to ensure complete lignin dispersion. Following this, the linker was added to the solution, for which α,α′-dichloroxylen (Sigma-Aldrich, 623-25-6) was selected. After the solubilization of the linker, the catalyst was added to the solution, with FeCl_3_ being used as the catalyst.

Synthesis was conducted using 1,2-dichloroethane as a solvent, anhydrous FeCl_3_ as a catalyst, and α,α′-dichloroxylen as a linker. The molar ratios were calculated based on the fact that the extracted lignin was primarily composed of coniferyl alcohol. The molar ratio of lignin:FeCl_3_:linker was 1:1.5:1.5. The reactions were conducted at 60 °C for 24 h. After this period, the reactions were halted with water, and the resulting solid polymers were filtered and washed with water to eliminate any discoloured water and ensure the complete removal of the catalyst. The obtained materials were then dried at 45 °C for 24 h.

### Apparatus

Infrared spectroscopy analyses were carried out using an IR Xross Fourier Transform Infrared Spectrophotometer (Shimadzu Corporation, Kyoto, JP) with 64 scans and a resolution of 4 cm^–1^ in a 4000–400 cm^–1^ spectral range.

N_2_ physisorption measurements were conducted at a temperature of 77 K within the relative pressure range of 10^–6^ to 1 P/P_0_, utilizing an Autosorb 1MP/TCD instrument manufactured by Quantachrome. Before analysis, the samples underwent outgassing (residual pressure *p* < 10–4 Torr) at 343 K for 3 h. The determination of specific surface areas was conducted by employing the Brunauer–Emmett–Teller equation (BET) within the residual pressure range from 0.05 to 0.25 P/P_0_. The pore size distributions were obtained by applying the NLDFT method (N_2_ on carbon (slit pore, NLDFT equilibrium model)). Thermogravimetric analyses (TGA) were conducted on a Setaram (Caluire, France) LABSYS evo (TGA, DTA/DSC) apparatus under N_2_ (gas flow rate: 40 mL/min), with 10 mg of samples being heated from 30 to 900 °C at a rate of 5 °C/min.

The UV–vis spectra were collected with UV–vis-NIR spectrophotometer Cary 5000 UV–vis-NIR spectrophotometer (Agilent, CA, USA) in the spectral range 200–800 nm. The adsorption process was followed by recording one spectrum every 5 s at a fixed wavelength of 585 nm.

^13^C cross-polarization magic angle spinning (CPMAS) NMR spectra were acquired on a Bruker Avance III 500 spectrometer (Bruker, MA, USA) equipped with a wide bore 11.75 T magnet, operating at frequencies of 500.13 MHz for ^1^H and 125.77 MHz for ^13^C. A 4 mm triple-resonance probe in double-resonance mode with MAS was used in all experiments. The samples were packed on a Zirconia rotor and spun at a MAS rate of 15 kHz. For the ^13^C CPMAS experiments, the radio frequency (RF) fields of 55 and 28 kHz were used for initial proton excitation and decoupling, respectively. During the CP period the ^1^H RF field was ramped using 100 increments, whereas the ^13^C RF fields were maintained at a constant level. During the acquisition, the protons are decoupled from the carbons by using a two-pulse phase-modulated (TPPM) decoupling scheme. A moderate ramped RF field of 62 kHz was used for spin locking, while the carbon RF field was matched to obtain optimal signal. The relaxation delay between accumulations was 1 s and the CP contact time was 2 ms. All chemical shifts are reported using δ scale and are externally referenced to tetramethylsilane (TMS) at 0 ppm.

## Results and Discussion

### Infrared Spectroscopy

Infrared spectroscopy was utilized to characterize the lignin derivative materials postsynthesis to enhance comprehension of the functional groups on the material’s surface. The obtained FTIR spectra facilitated the discernment of the materials in terms of OH groups, various CH_n_ groups, and other functional groups. The FTIR spectra are reported in Figure 1, in the high-frequency region between 4000 and 2750 cm^–1^, displaying two bands related to the intramolecular hydroxyl group at 3430 cm^–1^ and the intermolecular hydroxyl group at 3210 cm^–1^. Hydrogen bonds from the OH groups of the lignin fragment and some polyphenolic residuals drive these bonds. In the high-frequency region, between 3125 and 2750 cm^–1^, the stretching motion of the CH bond is evident, enabling the differentiation of the signals of the carbon–hydrogen bonds found in aromatic and aliphatic structures. Aromatic CH bonds show two peaks at 3042 and 3002 cm^–1^, corresponding to out-of-plane and in-plane stretching, respectively. These bonds are associated with lignin monomers and the employed linker. Aliphatic CH bonds can be detected at lower frequencies, with CH_3_ stretching at 2950 cm^–1^ and asymmetric and symmetric CH_2_ stretching at 2927 and 2846 cm^–1^, respectively. The CH_3_ and CH_2_ components are found in lignin monomers. Furthermore, a correlation between CH_2_ and the linker, similar to that observed for aromatic CH bonds, has been established.

**Figure 1:**
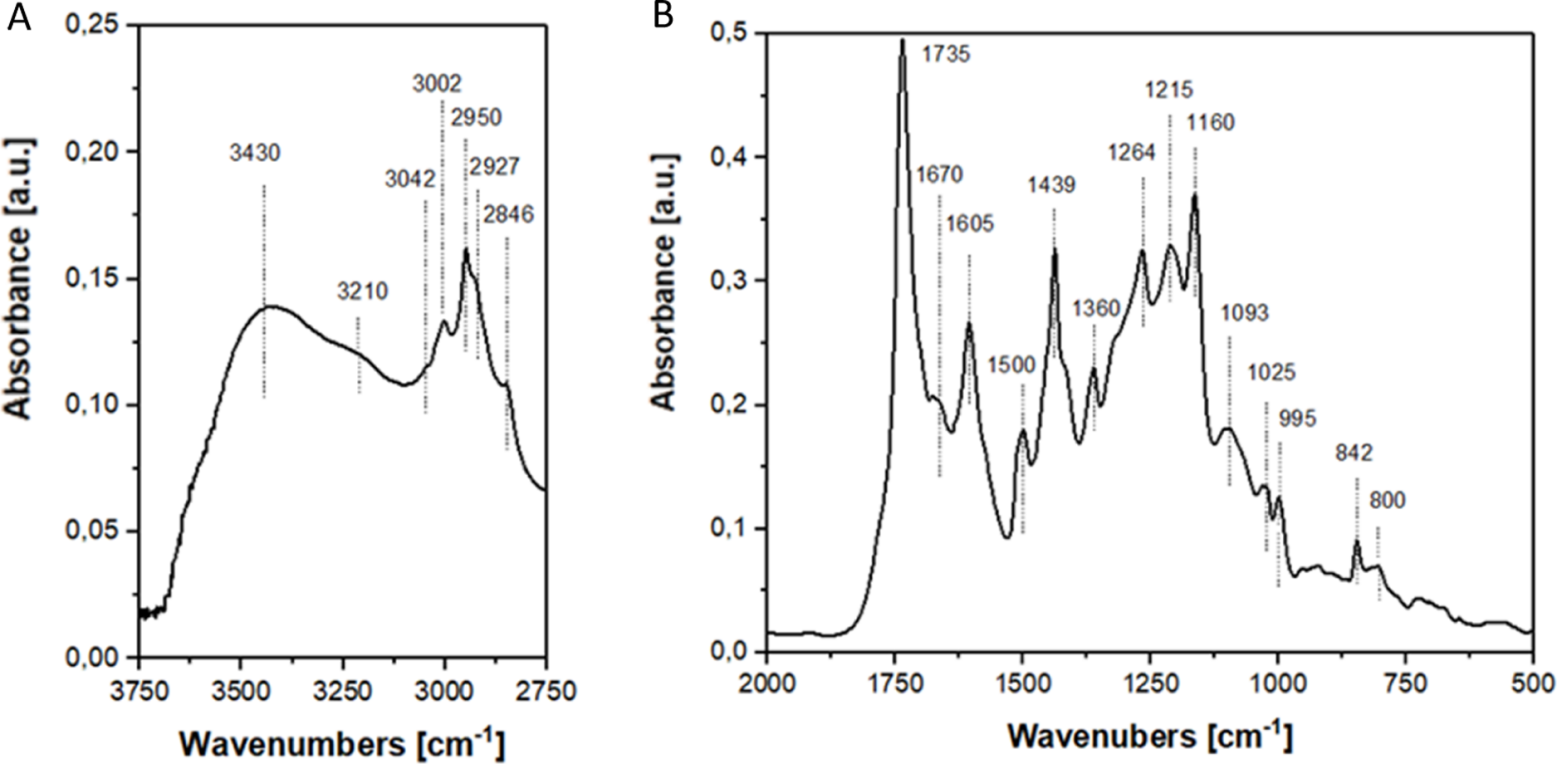
Infrared spectra of lignin-derivate material in the range 3750–2750 cm^–1^ (A) and 2000–500 cm^–1^ (B).

The skeletal motion of lignin-derived materials is observed in the lower frequency region, ranging from 2000 to 500 cm^–1^. Carbonyl groups are present at 1735 cm^–1^, exhibiting a very intense band associated with C=O stretching. Carbonyl groups are also present with two other bands: the first is related to the stretching of the C=O groups bonded to an aromatic ring and occurs at 1215 cm^–1^; the second one is associated with C–O stretching at 1093 cm^–1^. These two types of bonds are not related to the presence of carboxylic acids.

The peak at 1160 cm^–1^ is attributed to carbonyl groups, indicating the presence of ketonic groups in aliphatic short chains. The spectra also encompass the carbon–carbon skeletal motions associated with lignin monomers and linkers. Most peaks and bands relate to aromatic rings as skeletal motions at 1605 cm^–1^ or ring substitutions at 1500 and 842 cm^–1^. Most aromatic rings were substituted in ortho or para positions during synthesis, as indicated by a peak at 1500 cm^–1^. Only a tiny percentage of aromatic rings were substituted in meta, as reflected in the band at 800 cm^–1^. The peak at 842 cm^–1^ further supports this, and it relates to two hydrogen atoms positioned close to each other on an aromatic ring in the 2,4 substitutions. Carbon–carbon stretching is also apparent, with a peak at 1264 cm^–1^, which can be attributed to carbon–carbon single bonds within the structure.

In addition to the aromatic skeletal vibrations, specific peaks provide evidence of phenolic and pseudophenolic structures. The peak at 1670 cm^–1^ is ascribed to the characteristic double carbon–carbon bond of the phenolic structure, whereas the peaks at 1360 and 1317 cm^–1^ are linked to the bending of the carbon–hydrogen (CH) bond and to the presence of a substituent in the ortho position, respectively.

The peak at 1439 cm^–1^ is ascribed to the material’s lateral substituent, where CH_3_ characterizes in ether groups and symmetrical carbon–hydrogen stretching. In Table 1, the vibration motions noted in the FTIR spectrum and their assignments are reported.

**Table 1:** Assignment of the Vibrational Modes of the Lignin-Derived Material^49^

vibrational mode	frequency [cm^–1^]
ν OH intramolecular	3430
ν OH intermolecular	3210
ν_as_ CH aromatic	3042
ν_s_ CH aromatic	3002
ν_as_ C–H_3_-Ar	2950
ν_s_ C–H_3_-Ar	2927
ν_as_ CH_3_ aliphatic	2846
ν aliphatic ketone	1735
ν C=C–OH phenolic	1670
ν aromatic ring, skeletal vibration	1605
ν aromatic ring ortho or para mono substitute	1500
ν C=C bond to acceptor groups	1439
ν_s_ CH in OC–CH_3_	1360
δ COH in phenolic groups	1317
δ COH in o-phenol	1264
ν C–C + C–O	1215
ν Ar–C=O	1160
ν C-(C=O)-C of ketonic groups in aliphatic short-chain	1093
ν Ar–C–O	1025
ν benzene substituted, deformation out of plane	995
ν benzene substituted 1,4 or 1,2,5, def out of plane	842
ν benzene substituted 1,5 or 1,2,6, def out of plane	800

After preliminary characterization, the interaction between the material surface and a probe molecule was examined using infrared spectroscopy. Toluene was used as the probe molecule to investigate the interactions between a model pollutant and the material surface. Toluene is commonly used in the literature to assess the interactions of aromatic pollutants with the surfaces of various materials.^50–55^

As an aromatic molecule, toluene can simulate aromatic pollutants and act as a pollutant. In this case, it was employed to evaluate the interactions between aromatic compounds and the material surface. The toluene adsorption spectra from 38 to 10^–3^ mbar, compared to the toluene gas spectra, are shown in Figure 2.

**Figure 2:**
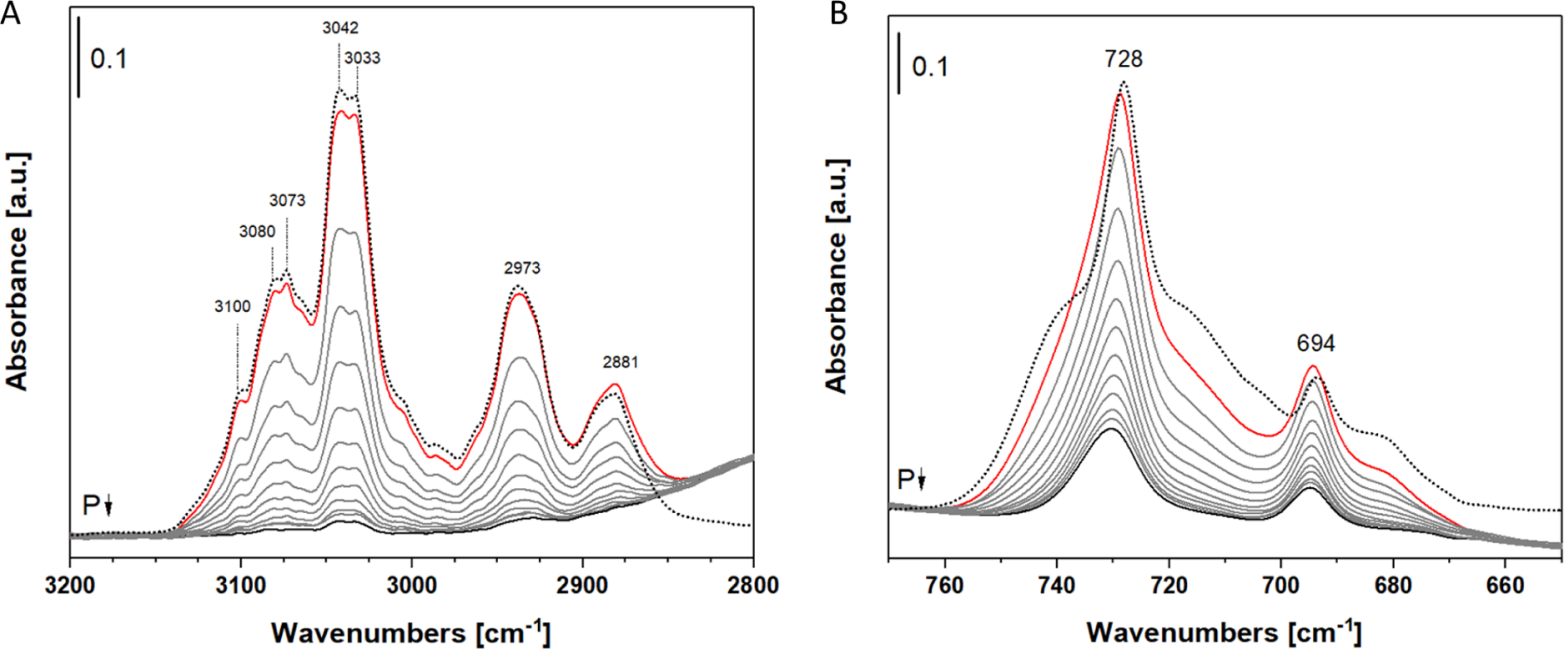
Infrared spectra of toluene adsorbed on lignin-based in the range 3200–2800 (A) and 770–650 cm^–1^ (B) materials at different pressures: spectra before adsorption of toluene in the gas phase (dashed curve), spectra after adsorption of 38 mbar (red curve) and subsequent desorption (final pressure <0.0018 mbar, black curve).

The toluene adsorption spectra of the synthesized materials exhibit a significant shift in the lower frequency region, ranging from 770 to 650 cm^–1^, where the stretching motions of the aromatic rings are observed. In the higher frequency range (3200–2800 cm^–1^), the stretching motions of both aromatic and methyl carbon–hydrogen bonds are evident.

As reported in Figure 2, a noticeable shift is apparent in the stretching bands of the aromatic toluene ring, indicating an interaction between the toluene molecules and the material. The figure displays toluene spectra at varying pressures, with the toluene bands associated with the symmetrical and asymmetrical vibrations of the carbon–hydrogen bonds of the CH_3_ group at 2875 and 2950 cm^–^^1^, respectively, whereas the vibrations of the carbon–hydrogen bonds of the aromatic ring occur at higher frequencies. No changes in the band positions are observed in this spectral region. This observation suggests that the methyl group of toluene does not interact directly with the material surface. In contrast, the area of the spectrum at lower frequencies, between 770 and 650 cm^–1^ (Figure 2B), shows a more pronounced variation in the position of the toluene bands between the gas phase and the adsorbed phase. The shift toward higher frequencies indicates the stabilization of the toluene aromatic ring on the surface of the material, suggesting a π–π interaction.^56^ To emphasize the shift in the toluene bands, the gas-phase contribution was subtracted from the adsorbed toluene; the postsubtraction spectra are presented in Figure 3. Following the subtraction, the aromatic ring vibration bands are particularly pronounced within the spectral region of 775 to 650 cm^–1^, which indicates the stabilization of the toluene molecules on the material’s surface. Conversely, in the spectral range between 3200 and 2800 cm^–1^, the carbon–hydrogen vibrations remain largely unaffected, with the contributions in this area attributed to the gas phase, resulting in negative bands postsubtraction.

**Figure 3:**
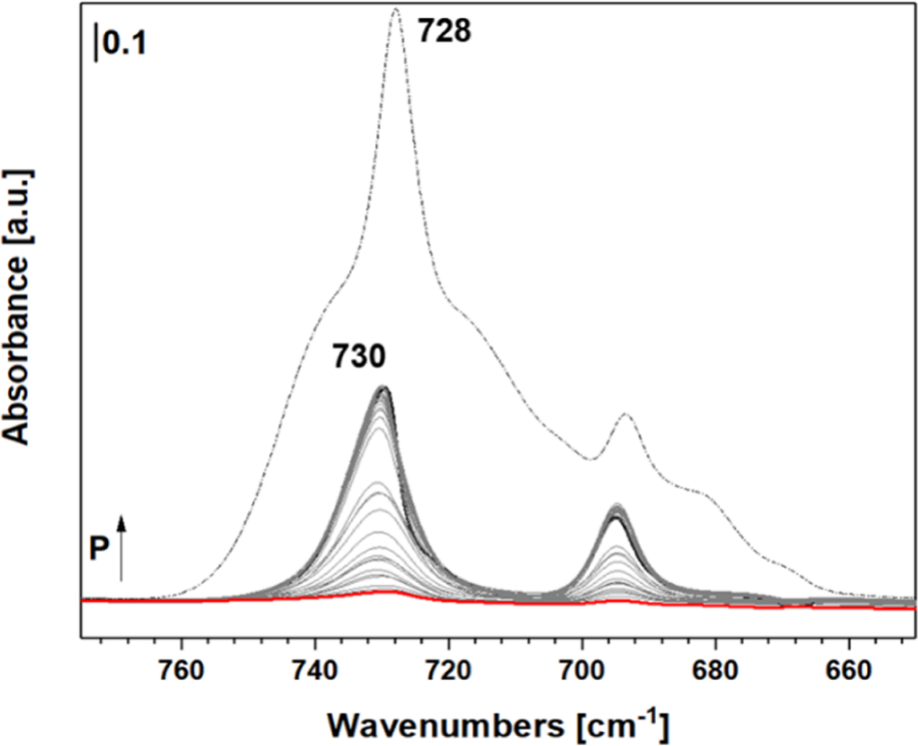
FTIR spectra of toluene adsorbed on lignin-derived materials in the range 775–675 cm^–1^ after subtraction of the gas phase contribution at 38 mbar (black curve) and subsequent desorption (final pressure <10^–3^ mbar, red curve) compared to the spectra of toluene gas phase (dashed curve).

The process of toluene adsorption was observed by tracking the intensity of the peak at 728 cm^–1^, which corresponds to the maximum of the most intense peak in the gas-phase spectra. The intensity of the peak at 728 cm^–1^ was monitored following the subtraction of the gas phase contribution; the resultant spectra are illustrated in Figure 4.

**Figure 4:**
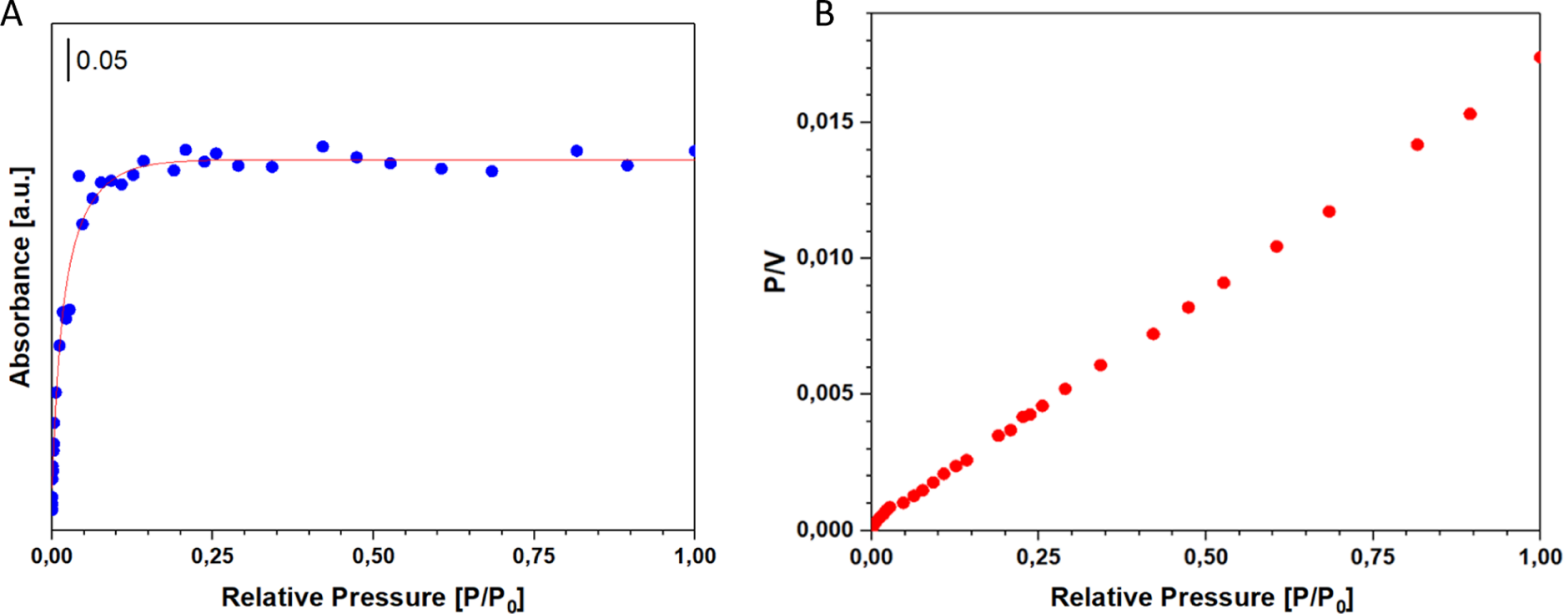
Optical isotherm (A) and linearization obtained by application of the Langmuir model (B) via the peak at 728 cm^–1^.

The spectra reported in Figure 3 indicate that only the stretching motions associated with the aromatic ring of the probe molecule are disturbed. In contrast, the stretching motions related to the methyl group of toluene remain unchanged. This observation is linked to the geometry of toluene packing during the adsorption process.

From the intensities of the spectra, after subtracting the contribution from the gas phase, it was possible to derive the absorbance values corresponding to the peak maximum at 728 cm^–1^ using the Lambert–Beer equation, allowing for the calculation of the number of molecules adsorbed onto the material’s surface. With the number of the adsorbed toluene molecules, an initial estimation of the surface area of the analyzed material was achievable. Below, Figure 4 illustrates the optical isotherms (Figure 4A) and the linearization by applying the Langmuir model (Figure 4B).

The surface area values of the material could be estimated using the optical isotherms. This estimation was assessed by linearizing the curves and extrapolating the adsorbed gas volume and surface affinity coefficients using the equations of the fitting lines. From the optical isotherm, it is possible to observe how, in the Langmuir range of relative pressure, the material is over the monolayer, and the calculation of the SSA is an estimation. The linearization, in the Langmuir range (0.1 < *P*/*P*_0_ < 0.3), allows for the evaluation of the material’s surface area.

All surface area calculation models used involve using the steric size values of the adsorbate molecules. Given the toluene structure, used as a probe molecule, this can interact with the surface via two different conformations. Either the toluene can interact with the surface of the material via interaction of the aromatic ring and the methyl group, positioning itself parallel to the surface, or it can interact via interaction of the ring perpendicular to the surface, changing the steric size of the toluene molecules on the surface. Due to the different sizes of the toluene molecules, different steric size values are present depending on the various arrangements of the molecules on the surface.^57^ Given the lack of interaction between the methyl group and the material surface, verifying the correct arrangement of the toluene molecules on the material surface was possible.

From the equations shown in Table 2, it was possible to calculate the surface area values of the synthesized materials. The model, as expected, differs in the calculated surface area values using N_2_ at 77 K. The size of toluene molecules, used for the calculation of SSA, is reported in the literature.^57^

**Table 2:** Toluene Adsorbed Volume, Coefficient *b*, and SSA Value Obtained by Applying Langmuir Model to the Infrared Spectra

model	Vm [ccSTP/g]	coefficient *b*	SSA [m^2^/g]
Langmuir	2942.4	0.02030	3409

### Porosimetric Analyses

In the realm of adsorbent materials, the parameters of surface area and porous volume hold significant importance. Consequently, porosimetric analyses were performed utilizing the physisorption of nitrogen (N_2_) at a temperature of 77 K. Through the evaluation of the resultant isotherms (refer to Figure 5), it became feasible to determine various surface area parameters and porous volumes. The BET (Brunauer–Emmett–Teller) equation was utilized to compute the surface area, with the range of relative pressures established between 0.05 and 0.25 *P*/*P*_0_. The measured surface area values for the synthesized materials were found to be 30 m^2^/g. Moreover, the isotherms facilitated the extrapolation of data related to pore volume and average pore diameter. The pore volume was 0.079 cc/g, while the average pore diameter was measured at 21.84 Å.

**Figure 5:**
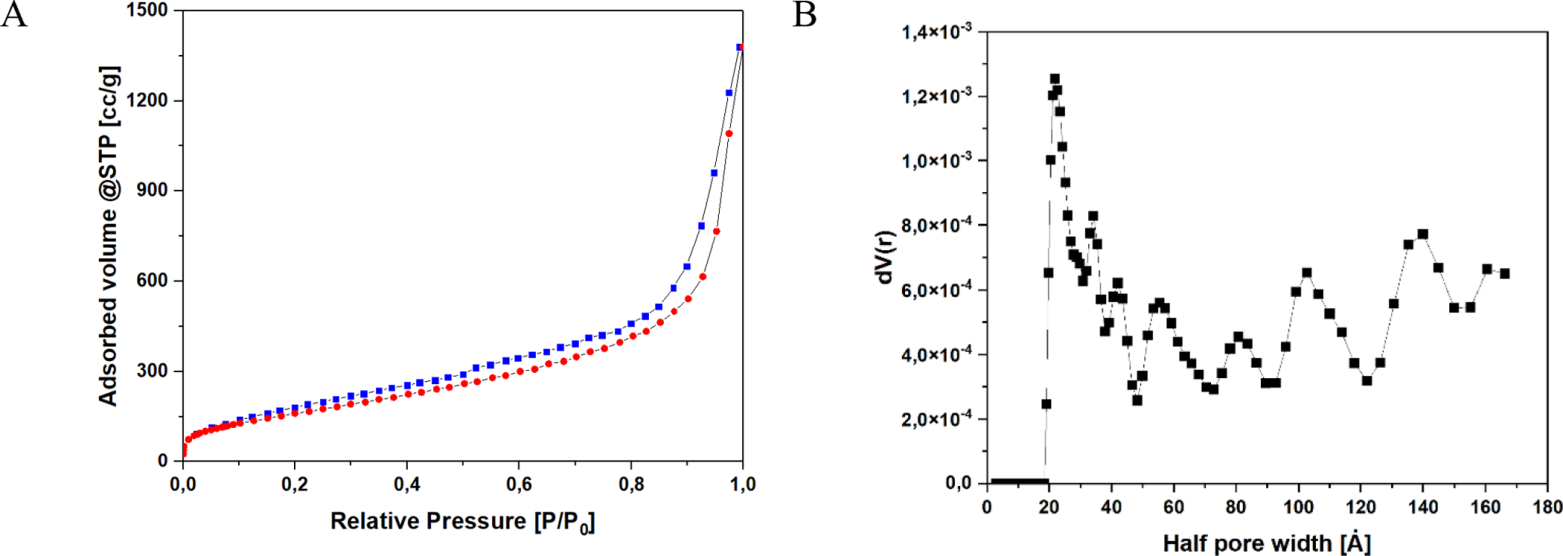
: N_2_ Isotherm adsorption at 77 K (A) and pore distribution and cumulative pore volume (B) of lignin-derived material.

The isotherms of the examined samples (Figure 5A) exhibited a considerable hysteresis loop, which persisted for *P*/*P*_0_ values below 0.4. This observation may suggest the materials’ increased flexibility and propensity to swell during adsorption. Figure 6B shows the distribution of the pores in the synthesized material. The porous distribution highlights that the synthesized material does not present a uniform pore family, but rather a family of pores, with different types of porosity and very different half pore between different from each other between 20 and 140 Å.

**Figure 6:**
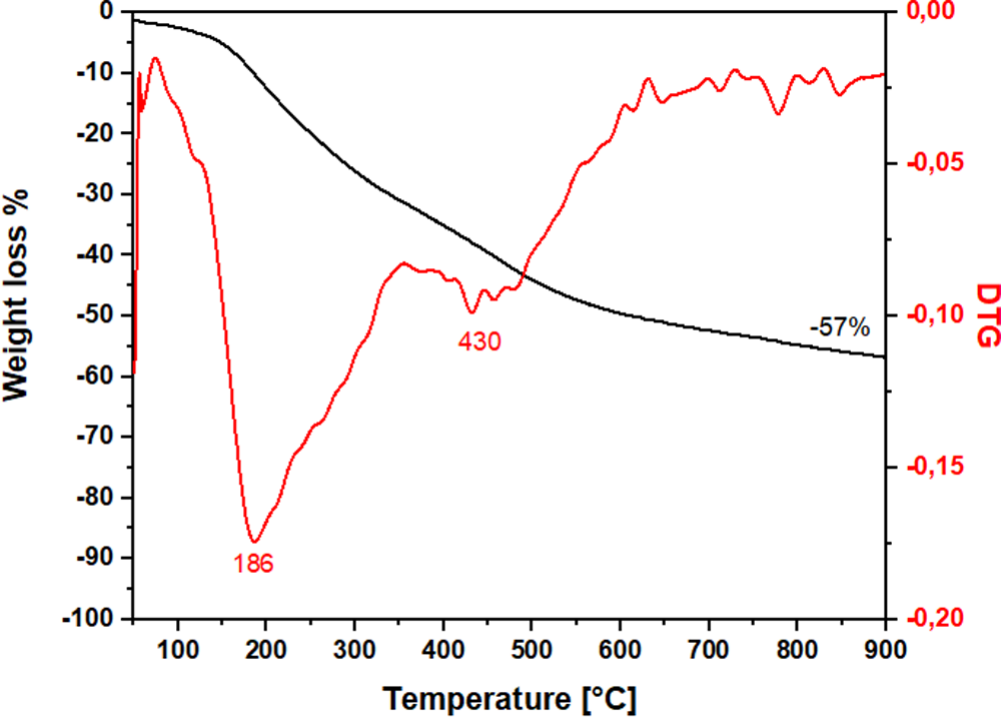
Thermogram (black curve) and first derivative (red curve) of lignin-based material.

### Thermogravimetric Analyses

Thermogravimetric analyses were conducted on the synthesized samples to evaluate their thermal stability; the results provided valuable insights into the extent of the materials’ conjugation.

Thermogravimetric analyses were conducted in N_2_ flow to assess the thermal stability of the obtained materials. The thermal profile of the samples revealed an initial weight loss at 110 °C, ascribed to the loss of physisorbed water and residual solvent within the material porosity. At 150 °C, the sample demonstrated a continuous and stable weight loss up to 900 °C. In the temperature range 150–900 °C, applying the first derivative profile enabled the identification of a weight loss at 340 °C, which relates to the thermal degradation of the lignin component of the material.^25^ In this temperature range, dehydration, demethylation, conjugation between aromatic rings, and loss of carboxyl and carbonyl groups occur.^58,59^ The percentage of residual material at 900 °C was 43% by weight, indicating a weight loss of 57%. The residual amount of materials at 900 °C is comparable to the residual weight of biochar produced by lignin.^60^ The thermogram of the obtained materials is reported in Figure 6.

### Nuclear Magnetic Resonance

NMR characterization is a widely used technique for analyzing lignin.^61,62^ In this work, solid-state ^13^C NMR spectroscopy was used to investigate the differences in terms of structure and functional groups in lignin-based materials after the cross-linking reaction. ^13^C CPMAS NMR spectra of the extracted lignin and lignin-based biopolymer-composite material are shown in Figure 7.

**Figure 7:**
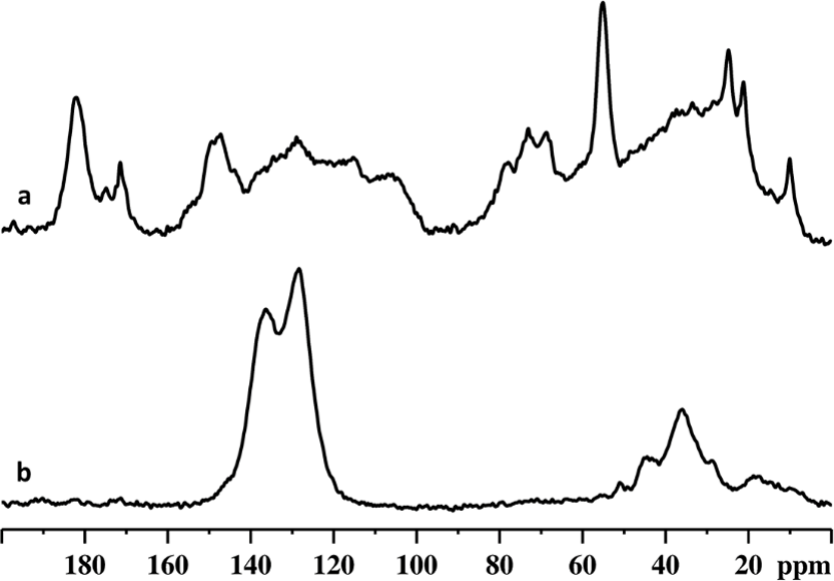
^13^C CPMAS NMR spectra of extracted lignin (a) and lignin-based material (b).

The extracted lignin sample (Figure 7a) have characteristic ^13^C NMR peaks associated with lignin carbons. The most intense peaks in the spectrum are due to methoxy and aromatic lignin carbons. However, overlapping ^13^C resonance peaks corresponding to the presence of trace amounts of cellulose and hemicellulose were also detected.^63^

The ^13^C CPMAS NMR spectrum of lignin-based material synthesized by Friedel-Craft reaction, reported in Figure 7b, displayed intense peaks associated with aromatic groups, specifically at 128 and 136 ppm, due to aromatic carbons with C–H groups and substituted aromatic carbons, respectively.^64^ The ^13^C spectrum contains superimposed contributions of aromatic carbons from dichloroxylene and lignin. This overlap makes it difficult to clearly distinguish the two contributions in such a ^13^C CPMAS spectrum. Moreover, a peak centered at 36 ppm is detected in the spectrum and is assigned to carbons associated with methylene groups cross-linking dichloroxylene and lignin. Additional ^13^C low-intensity peaks are detected at 18, 28, 45, and 55 ppm that are related to fragments such as methyl, ethyl, −CH_2_Cl and methoxy groups, respectively.^65^ Only very weak signals, attributable to cellulose and hemicellulose, are present in the ^13^C spectrum, suggesting that they have been successfully eliminated in the lignin-based material.

### UV–Vis Spectroscopy

UV–vis spectroscopy is a widely used technique to analyze the reaction kinetics of chemical species. In this instance, the reaction kinetics of the Crystal Violet dye in an aqueous solution were monitored. This dye has a highly characteristic absorption spectrum in the visible light range. By tracking the variation in the intensity of the absorption maximum, the quantity of dye adsorbed by the material over time could be observed. The decision to use an aromatic dye such as Crystal Violet was suggested by the π-π interactions observed through FTIR spectroscopy. The presence of π–π interactions between an aromatic molecule and the material was verified using a dye with aromatic structures, as shown in Figure 8A, and was also verified through UV–vis spectroscopy.

**Figure 8:**
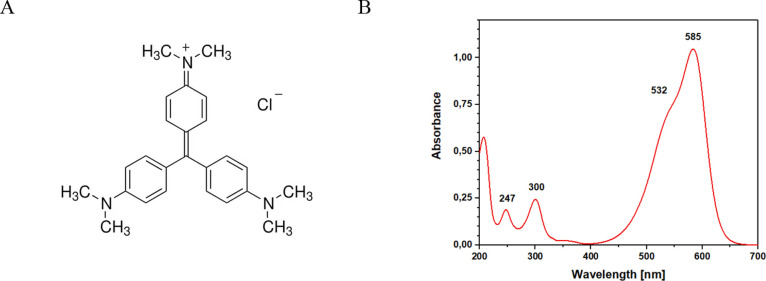
: Crystal violet molecular structure (A) and UV–vis spectra of Crystal violet solution (B).

The dye adsorption by the material was monitored continuously, with a single data point recorded every five seconds. The analysis wavelength was determined to be 585 nm, which corresponds to the absorption maximum of Crystal Violet, as shown in Figure 8. The maximum at 585 nm corresponds to electronic transitions π–π*; the same transition occurs at 532 nm, and the presence of Crystal Violet isomers causes it.^66^ Two bands in the UV region at 300 and 247 nm are associated with n-π* transition of a nitrogen electron and π–π* transitions of the delocalized electrons in the aromatic rings.^67^

Following the establishment of the analysis wavelength, the change in light absorption was measured over the initial 5 min of the process, which remained constant. Subsequently, the material was introduced to the solution (comprising 0.25% of the solution’s mass) and agitated continuously throughout the adsorption process. The actual adsorption kinetics of the dye on the material could be determined by maintaining a constant flow of the solution within the measuring cell. The resultant data (reported as √*t*, and one point every 1 min), reported in Figure 9, indicate the change in the corresponding peak at 585 nm of the dye as a function of √time.

**Figure 9:**
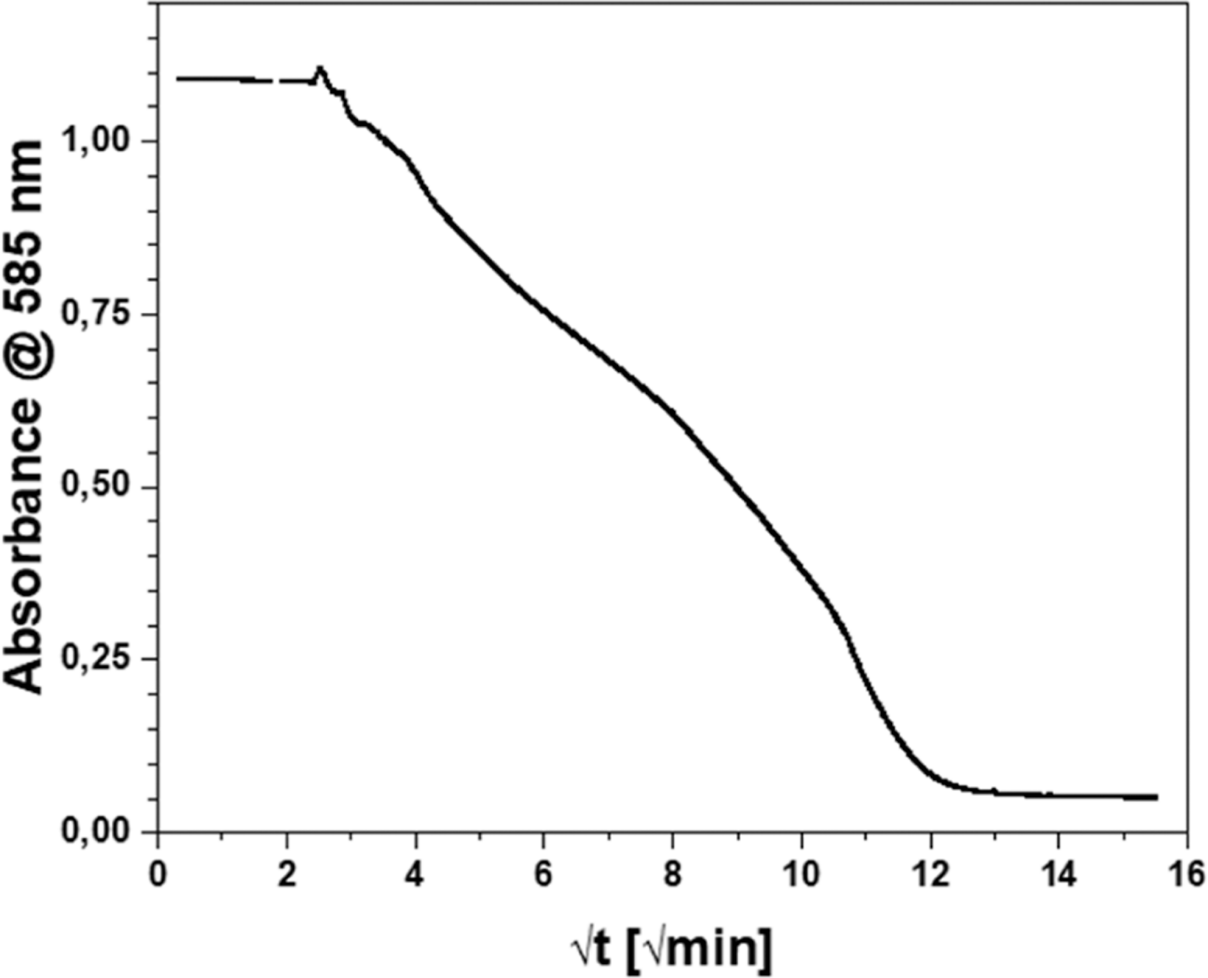
Absorbance variation of the crystal violet solution during the adsorption process on lignin-based material; the curve reports the absorbance values from 0 to 240 min, expressed as √*t*.

The adsorption curve of the Crystal Violet dye on the synthesized material displayed four distinct zones, each characterized by variations in the slope of the curves, which correspond to the following time intervals: 15–40, 41–120, 121–160, and 161–240 min. The first 15 min were removed from the kinetics calculation, as in the first 5 min the system was kept stable until a constant absorbance value was maintained, and then the material was added. The 5 min following the addition of the material were removed from the kinetics calculation to allow the material to disperse homogeneously in the solution. The next 5 min (from 10 to 15 min) were excluded from the kinetic calculation in order to remove the interference of the small particles that can cause scattering phenomena. During the initial phase (15–40 min), a rapid decrease in the slope of the curve was evident, with this decline occurring within moments of contact. Subsequently, in the second time range (41–120 min), a significant change in the slope of the curve was observed, indicating a distinct dye adsorption onto the material. In the third step (121–160 min), the curve underwent a shift in slope, assuming a value comparable to the initial one, characterized by accelerated dye adsorption relative to the preceding step. The solution’s absorbance value stabilized in the final range, spanning from 161 to 240 min, with no discernible adsorption process occurring.

The observed changes in the slope of the curves during the specified time frames suggest a modulation in the material’s underlying dye adsorption mechanism. Additionally, a change in the hydrophilicity of the material was observed, which increased in proportion to the amount of the adsorbed dye. The most significant change in slope, occurring between 41 and 120 min, could be attributed to the diffusion of the dye within the material particles following interaction with the surface. At this stage, the diffusion process appeared slower than the adsorption process, resulting in a progressive decrease in the adsorption rate. The result of the adsorption of the dye by the material was a 98% decrease in the value of the solution’s absorbance. To enhance understanding of the mechanisms involved in these phases, the equations that most accurately describe the progression of the curves were calculated. Figure S1 in the Supporting Information, shows the ranges of the adsorption process as a function of √*t* alongside the curve that best fits the experimental data. The displayed curves demonstrate nonlinear trends and vary throughout the dye adsorption process. The first interval (Figure S1A) illustrates how the adsorption phenomenon is characterized by an allometric function, in which the dye adheres to the material’s surface exposed to the aqueous solution. In the second interval (Figure S1B), the curve displays an exponential decay trend, emphasizing the exponential dependence of time on the adsorption process. This phase is the longest and determines the overall dye adsorption kinetics. In the final part of the curve (Figure S1C), the adsorption process exhibits a logarithmic trend, which correlates with the increase in hydrophilicity of the material caused by the presence of the adsorbed dye molecules. Through various interpolations of the curves, it was possible to extrapolate the kinetic constants of adsorption for each process phase. In the first step, the adsorption constant exhibits the highest value, indicating that the process rapidly progresses during this phase. In the second step, the adsorption constant reaches approximately half that of the first step, suggesting a decrease in the dye adsorption rate onto the material compared to the first step. In the final step, the adsorption constant achieves a minimum value. The kinetic equations and the values of the constants are presented in the Supporting Information Table S1. In Table S2, for comparison, are reportered the kinetic model and the adsorption kinetic constant known in literature for other materials.

Saturation tests were carried out using the materials to ascertain the maximum adsorption capacity of Crystal Violet. These tests replicated the experimental conditions of adsorption kinetics with the material in contact with a saturated dye solution. The material was left in contact with a 4.0 g/L Crystal Violet solution for 24 h. Subsequently, the material was separated from the solution, and the quantity of adsorbed dye was measured gravimetrically. The tests indicated that the material’s maximum dye adsorption was 1.8 g of dye per g of material, corresponding to 66 wt %. The amount of dye needed to saturate the materials is compared with the common materials used for adsorption. The data available in the literature regarding Crystal Violet from a water solution are reported and compared in Table 3.

**Table 3:** Comparison between the Adsorption Capacity of the Materials for Crystal Violet Removal

materials	adsorbed dye [g/g]	reference
Arabic gum-cl-poly(acrylamide) hydrogel	0.10	^68^
organo-clay	0.16	^69^
carbon materials	0.40	^70^
graphene oxide-montmorillonite composites	0.75	^68,71^
chitin/ZSM5	1.22	^72^
this work	1.86	-

Data reported in Table 3 shows different materials commonly used in literature as sorbent materials for Crystal Violet. Comparing the adsorption capacity of the different classes of materials with the lignin-based material synthesized in this work, it is possible to note the higher capacity under saturation. The higher adsorption capacity is due to the ability of the material to swell and increase the number of points where the aromatic ring of the dye can interact with the lignin aromatic ring.

## Conclusions

Lignin was extracted from micronized bamboo culms under alkaline conditions and subsequently repolymerized using α,α′-dichloroxylen as a linker via a Friedel–Crafts alkylation reaction. The synthesized materials underwent comprehensive characterization through a range of analytical techniques. Infrared spectroscopy was used to ascertain the present surface functional groups. The infrared spectra provided information about the resulting polymeric materials’ surface characteristics and aromatic framework. FTIR spectroscopy assessed the interactions between the synthesized materials and toluene as a model pollutant. By conducting FTIR measurements at different toluene pressures, we could observe the interactions between the aromatic rings of the probe molecules and the functional groups of the surface of the materials. Moreover, from the FTIR spectra of toluene, it was possible to estimate the specific surface area of the material. During the calculation phase, it was feasible to ascertain the configuration of toluene on the material’s surface. A perpendicular interaction between the surface of the material and the aromatic ring was identified, characterized by the positioning of the methyl groups at a relevant distance from the surface, resulting in no perturbation. Essential parameters, including surface area, pore volume and pore diameter, were evaluated within the context of adsorbent material utilized for pollutant capture. Porosimetric analysis indicated that the material exhibits a low surface area (30 m^2^/g), along with reduced pore volume and pore diameter. Nevertheless, the isothermal data indicated the material’s structural adaptability, as evidenced by the absence of a precise hysteresis loop closure. Moreover, the specific surface area calculated from the isotherm indicates the low affinity between nitrogen and the material’s surface, if compared to toluene adsorption. Additionally, thermogravimetric analysis revealed that the thermal degradation profile of the synthesized material closely resembles that of lignin, occurring within the 350–500 °C temperature range. Furthermore, weight loss was observed at lower temperatures, likely linked to the entrapment of solvents within the material’s porosity. ^13^C CPMAS NMR data showed the difference between the extracted lignin and the lignin-based material. The superimposed contributions of aromatic carbons from dichloroxylene and lignin in the ^13^C NMR spectrum confirmed the successful cross-linking occurred during the Friedel–Crafts alkylation reaction. Despite surface area measurements being lower than those of commonly employed adsorbents for pollutant removal, we conducted removal tests using Crystal Violet dye as a model aromatic contaminant. The adsorption dynamics were continuously assessed, focusing on the peak intensity of Crystal Violet at 585 nm. Samples were collected every 5 s over 4 h to analyze the kinetics of the adsorption process. The results indicated that the adsorption did not follow a linear progression; instead, it evolved in complexity over time. The material’s capacity to adsorb large quantities of dye is directly related to its ability to swell upon interaction with an aromatic compound, thereby compensating for the low specific surface area measured by N_2_ physisorption. Saturation tests demonstrated an adsorptive capacity of 1.8 times by weight, confirming that lignin-derived materials are viable candidates for removing aromatic pollutants.

## Supplementary Material



## Notes

Extrapolation of kinetic equations for the first, second, and third steps in the adsorption process (Figure S1); kinetic equations of the different processes of dye adsorption on the surface of the material (Table S1); and adsorption capacity, removal efficiency, kinetic model, and kinetic constant, operative conditions for the Crystal Violet removal materials (Table S2) (PDF)
