# Supplementary material for: Synthesis and Characterization of Lignin-Derived Porous Materials from Phyllostachys edulis (Bamboo Moso) for the Removal of Aromatic Pollutants
Source: ACS Omega. 2025 Nov 17;11(11):17137–47. doi: 10.1021/acsomega.5c06041 (PMC13019262; doi:10.1021/acsomega.5c06041)
Supplement: Supplementary file 1 [file ao5c06041_si_001.pdf]

***Synthesis and Characterisation of Lignin-Derived Porous Materials* from *Phyllostachys Edulis* (Bamboo Moso) for the Removal of Aromatic Pollutants**

Andrea Marangon[1,2], Elisa Calà[1,2*], Alessandro Croce[3], Geo Paul[4], Giorgio Gatti[1,2]

[1]Dipartimento per lo Sviluppo Sostenibile e la Transizione Ecologica, Università degli Studi del Piemonte Orientale, Piazza Sant'Eusebio 5 - 13100 Vercelli, Italy
[2]GEA G.S. s.r.l.s., Piazza S. Eusebio 5 - 13100 Vercelli, Italy
[3] SSD Research Laboratories, Research and Innovation Department (DAIRI), Azienda *Ospedaliero-Universitaria SS. Antonio e Biagio e Cesare Arrigo, Via Venezia 16, 15121 Alessandria, Italy*
[4]Dipartimento di Scienze e Innovazione Tecnologica, Università degli Studi del Piemonte Orientale, Viale Teresa Michel 11 - 15121 Alessandria, Italia

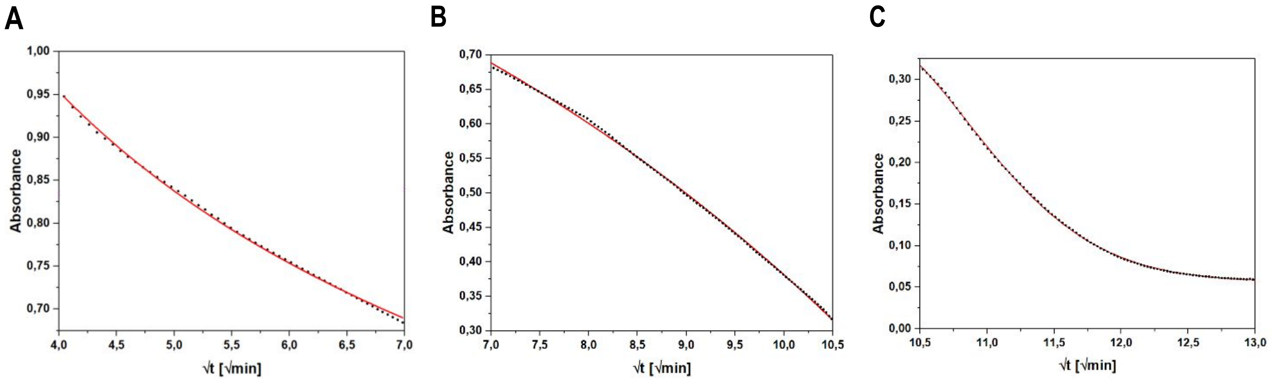

Figure S1: Extrapolation of kinetic equations for the first (A), second (B), and third (C) steps in the adsorption process

Step	Interval [√min]	Equation	R^2	K
1°	4-7	$y = 2.13\,x^{-0.58}$	0.99883	2.13
2°	7-10.5	$y = -0.197^{-(x/6.78)} + 1.24$	0.99957	1.24
3°	10.5-13	$y = 0.056 + \left\{ \frac{0.655}{\left((\sqrt{2\pi}) \cdot 0.084x\right)^{\left(-\ln((x)/10.073)^{2/(2\cdot 0.084^2)}\right)}} \right\}$	0.99985	0.06

Table S1: Kinetic equation of the different processes of dye adsorption on the surface of the material

Material	Adsorption Capacity (mg/g)	Removal Efficiency (%)	Kinetics Model	Kinetic costant	Operational Parameters Influence	Ref.
Zeolite-embedded carboxymethyl tamarind kernel gum hydrogels	123,6	Not specified	Pseudo-first-order	0.0049 min^{-1}	pH varied	1
Fe_3O_4 nanoparticles-coated biochar	349,4	>90	Pseudo-first and second order	0.870 $L\ mg^{-1}$	pH not specified, 55°C	2
Ordered Mesoporous Zr-Ce-SBA-15 Composites	105,0	93.87	Pseudo-second-order	0.035 $L\ mg^{-1}$	pH 3, 20 min, 45 °C	3
Biochar prepared from coconut shell	124,5	Not specified	Pseudo-second-order	0.24153 $g\ mg^{-1}\ min^{-1}$	Pyrolysis at 700 °C	4
Anionic polymer brush functionalized mesoporous silica nanoparticles	128,4	91	Pseudo-second-order	0.0076 $L\ mg^{-1}$	pH 3, 100 ppm	5
Mechanically Activated Talc	160,0	Not specified	Pseudo-second-order	0.0000162 $g\ mg^{-1}\ min^{-1}$	pH and grinding effects	6
Polymeric Membrane from breadfruit peel	176,3	Not specified	Pseudo-second-order	0.006 $L\ mg^{-1}$	pH 11, 150 min	7
Natural Clay	206,7	Not specified	Pseudo-second-order	0.0310 $L\ min^{-1}$	pH 7.2, 23-45 °C	8
Activated Carbon/$ZnFe_2O_4$ Nanocomposite	246,2	Not specified	Pseudo-second-order	1.34 $L\ mg^{-1}$	pH 6, 80 min, 25°C	9
Graphene oxide-ED@Cellulose	260,0	>98	Pseudo-second-order	0.011 $g\ mg^{-1}\ min^{-1}$	Citric acid functionalization	10
Phosphate-rich polyacrylonitrile fiber	354,5	>90	Pseudo-second-order	3.485 $g\ mmol^{-1}\ min^{-1}$	25 min, pH not specified	11
Activated Carbon	428,0	98	Pseudo-second-order	0.34 $L\ min^{-1}$	55 °C, high bentonite content	12

Table S2: Adsorption capacity, removal efficiency, adsorption model, and adsorption kinetic constants and conditions of crystal violet removal materials

References:

(1) Rani, I.; Warkar, S. G.; Kumar, A. Removal of Cationic Crystal Violet Dye Using Zeolite- Embedded Carboxymethyl Tamarind Kernel Gum (CMTKG) Based Hydrogel Adsorbents. *ChemistrySelect* **2023**, *8* (29), e202301434. https://doi.org/10.1002/slct.202301434.

(2) Sun, P.; Hui, C.; Azim Khan, R.; Du, J.; Zhang, Q.; Zhao, Y.-H. Efficient Removal of Crystal Violet Using Fe3O4-Coated Biochar: The Role of the Fe3O4 Nanoparticles and Modeling Study Their Adsorption Behavior. *Sci Rep* **2015**, *5* (1), 12638. https://doi.org/10.1038/srep12638.

(3) Xiang, G.; Long, S.; Dang, A. Fabrication of the Ordered Mesoporous nZVI/Zr-Ce-SBA-15 Composites Used for Crystal Violet Removal and Their Optimization Using RSM and ANN–PSO. *Sustainability* **2022**, *14* (11), 6566. https://doi.org/10.3390/su14116566.

(4) Rodrigues, J.; Shetye, S.; Raju, S. Removal of Crystal Violet, an Emerging Pollutant from Aqueous Solution Using Biochar Prepared from Coconut Shell. *J. ISAS* **2024**, *2* (4), 74–90. https://doi.org/10.59143/isas.jisas.2.4.HFXO7613.

(5) Alswieleh, A. Adsorption Equilibrium and Kinetics Studies of Crystal Violet Adsorbed by Anionic Polymer Brush Functionalized Mesoporous Silica Nanoparticles. *Mater. Res. Express* **2023**, *10* (12), 125004. https://doi.org/10.1088/2053-1591/ad1315.

(6) Afifi, S.; Farhat, M.; Abdel-Khalek, M.; El-Dars, F. Removal of Crystal Violet and Acid Red 1 Dyes from Wastewater by Mechanically Activated Talc. *International Journal of Materials Technology and Innovation* **2023**, *0* (0), 0–0. https://doi.org/10.21608/ijmti.2023.244260.1097.

(7) Radoor, S.; Jayakumar, A.; Karayil, J.; Kim, J. T.; Siengchin, S. Biodegradable Polymeric Green Adsorbent for the Highly Efficient Removal of Crystal Violet Dye from Aqueous Solution. *Chemical Engineering Research and Design* **2023**, *199*, 473–485. https://doi.org/10.1016/j.cherd.2023.09.048.

(8) Alorabi, A. Q.; Hassan, M. S.; Alam, M. M.; Zabin, S. A.; Alsenani, N. I.; Baghdadi, N. E. Natural Clay as a Low-Cost Adsorbent for Crystal Violet Dye Removal and Antimicrobial Activity. *Nanomaterials* **2021**, *11* (11), 2789. https://doi.org/10.3390/nano11112789.

(9) Algarni, T. S.; Al-Mohaimeed, A. M.; Al-Odayni, A.-B.; Abduh, N. A. Y. Activated Carbon/ZnFe2O4 Nanocomposite Adsorbent for Efficient Removal of Crystal Violet Cationic Dye from Aqueous Solutions. *Nanomaterials* **2022**, *12* (18), 3224. https://doi.org/10.3390/nano12183224.

(10) Zarrik, B.; El Amri, A.; Bensalah, J.; Jebli, A.; Lebkiri, A.; Hsissou, R.; Hbaiz, E. M.; Rifi, E. H.; Lebkiri, A. Adsorption of Crystal Violet Using a Composite Based on Graphene Oxide-ED@Cellulose: Adsorption Modeling, Optimization and Recycling. *Inorganic Chemistry Communications* **2024**, *162*, 112179. https://doi.org/10.1016/j.inoche.2024.112179.

(11) Xu, G.; Jin, M.; Wang, F.; Kalkhajeh, Y. K.; Xiong, Q.; Zhang, L.; Tao, M.; Gao, H. Construction of a Phosphate-Rich Polyacrylonitrile Fiber Surface Microenvironment for Efficient Purification of Crystal Violet Wastewater. *RSC Adv.* **2019**, *9* (64), 37630–37641. https://doi.org/10.1039/C9RA07199G.

(12) Mulla, B.; Ioannou, K.; Kotanidis, G.; Ioannidis, I.; Constantinides, G.; Baker, M.; Hinder, S.; Mitterer, C.; Pashalidis, I.; Kostoglou, N.; Rebholz, C. Removal of Crystal Violet Dye from Aqueous Solutions through Adsorption onto Activated Carbon Fabrics. *C* **2024**, *10* (1), 19. https://doi.org/10.3390/c10010019.
